# Revisiting interaction in knowledge translation

**DOI:** 10.1186/1748-5908-2-34

**Published:** 2007-10-30

**Authors:** Liane R Ginsburg, Steven Lewis, Lisa Zackheim, Ann Casebeer

**Affiliations:** 1 School of Health Policy & Management, Faculty of Health, York University, 4700 Keele Street, Toronto, Ontario, M3J1P3, Canada; 2 Access Consulting, Saskatoon, Saskatchewan, Canada; 3 Department of Psychology, Faculty of Health, York University, 4700 Keele Street, Toronto, Ontario, M3J1P3, Canada; 4 Department of Community Health Sciences, Faculty of Medicine, University of Calgary, Alberta, Canada

## Abstract

**Background:**

Although the study of research utilization is not new, there has been increased emphasis on the topic over the recent past. Science push models that are researcher driven and controlled and demand pull models emphasizing users/decision-maker interests have largely been abandoned in favour of more interactive models that emphasize linkages between researchers and decisionmakers. However, despite these and other theoretical and empirical advances in the area of research utilization, there remains a fundamental gap between the generation of research findings and the application of those findings in practice.

**Methods:**

Using a case approach, the current study looks at the impact of one particular interaction approach to research translation used by a Canadian funding agency.

**Results:**

Results suggest there may be certain conditions under which different levels of decisionmaker involvement in research will be more or less effective. Four attributes are illuminated by the current case study: stakeholder diversity, addressability/actionability of results, finality of study design and methodology, and politicization of results. Future research could test whether these or other variables can be used to specify some of the conditions under which different approaches to interaction in knowledge translation are likely to facilitate research utilization.

**Conclusion:**

This work suggests that the efficacy of interaction approaches to research translation may be more limited than current theory proposes and underscores the need for more completely specified models of research utilization that can help address the slow pace of change in this area.

## Background

There has been increased emphasis on the topic of research utilization over the recent past [1-5]. Research utilization, discussed more now in the context of knowledge translation, has also become a priority for healthcare research funding bodies internationally [6], the UK's Medical Research Council, and one Canadian research foundation has dedicated itself to knowledge transfer [7]. Science push models that are researcher driven and controlled and demand pull models emphasizing users/decisionmaker interests have largely been abandoned in favour of more interactive models that emphasize linkages between researchers and decisionmakers [8]. However, despite these and other theoretical and empirical advances in the area of research utilization, there remains a fundamental gap between the generation of research findings and the application of those findings in practice. In order to begin filling this gap, we build on work that recognizes that research findings are used in many ways and have multiple effects [9-11]. Using a case approach, the current study looks at the impact of one particular interaction approach to knowledge translation used by a Canadian funding agency. By specifying some of the conditions under which different levels of decisionmaker involvement in research might be more or less effective, this work suggests that the efficacy of interaction approaches to research translation may apply to more limited contexts than what is prescribed by current theory. Note that the terms research utilization, knowledge utilization and knowledge translation are used throughout this paper in keeping with the terminology used by the authors we cite. These processes are roughly equivalent though RU is sometimes seen as narrower than KT. In this paper we are referring to the process whereby research is transferred and used (either instrumentally, conceptually or symbolically, read further for an explanation of these terms).

While the area of knowledge translation extends well beyond the life of any given study [7], ensuring that findings from certain individual research studies have an impact remains an important area for research. Our review of the literature focuses on what is known about effective approaches to research utilization, and we draw on the broader field of knowledge translation and the organizational literature to the extent that findings in these areas add additional insight.

Landry, *et al*. describe several models of knowledge utilization including the push model (emphasizing research production), the pull model (marked by user driven research), and the dissemination model that focuses on the dissemination effort itself [8]. The fourth model they describe, the interaction model, is said to successfully address the shortcomings of the other three models and proposes a linkage variable or mechanism that brings researchers and decisionmakers together throughout the research process. Landry *et al*. suggest that "the more resources the researchers invest in these types of linkage mechanisms, the higher the use of social science research" [8]. Indeed, the absence of interactions between researchers and decisionmakers has been cited as the primary reason for low utilization of research findings [12], and interaction approaches to research utilization are perceived to be the most valuable approaches by decisionmakers according to a recent systematic review [13].

In terms of the nature of researcher-decisionmaker interactions, the literature highlights the importance of face-to-face contact and interaction through forums that bring researchers and decisionmakers together to facilitate interpretation of research results [5,14,15]. In the organizational literature, joint interpretive forums to discuss and interpret study results were shown to have a positive relationship to the perceived usefulness of a research project [4]. More general forums for sharing research knowledge have also been proposed as one element of a 'communicative perspective' on research collaboration [16].

The literature is also fairly definite on the span or duration of these interactions. Lomas notes that "the clearest message from evaluation of successful research utilization is that early and ongoing involvement of relevant decisionmakers in the conceptualization and conduct of a study is the best predictor of its utilization" [17]. The importance of involvement beginning in the initial stages of a research study (*e.g*. the development of research questions) is central to participatory and community based research [18] and other work on researcher-decisionmaker interactions in healthcare [15,19] and education [14]. The presence of a historical or longstanding relationship between researchers and decisionmakers, where research utilization is only one activity in a larger, ongoing relationship, has been identified as important for the utilization of research findings [1,4,7,16]. This is consistent with political science and sociological perspectives, with the organizational literature, and work in the larger field of knowledge translation and knowledge creation, suggesting that both research utilization and knowledge translation are highly social processes that are more successful in the presence of positive social interactions between communities [3,20,21].

In fact, it is often suggested that relationships and face-to-face contact are more important to effective research utilization than the quality, methods, content of a research study, or its 'fit' with a decisionmaker's expressed need for the research [8,11]. This has to do with the fact that the determinants of research utilization are often organizational or political [22], and only rarely rational [17]. Two recent empirical studies found that interaction between researchers and decisionmakers, which began at the planning stage of a study, did not influence utilization of study results. Interestingly, in one of the studies this occurred because study findings were in conflict with decisionmaker's organizational and political interests [22]. In the other study, where findings confirmed existing practice, applied use was found to occur equally across groups that were involved in the planning and interpretation of the research and groups that were not involved [23]. These observations are entirely consistent with literature on the use of information in decision-making in the organizational literature [24-26]. For instance, information or research findings that are consistent with our values or expectations tend to be accepted, while information that is inconsistent tends to be challenged, questioned, and ultimately disregarded [22,27]. Accordingly, it has been argued that the importance of alignment between research findings and institutional context should not be underestimated [28,29].

Ross, *et al*., in their qualitative study of researcher-decisionmaker partnership experiences, broaden our understanding of the nature of decisionmaker involvement in research by articulating three models: first, formal support – where decisionmakers explicitly support, facilitate access to resources, and confer legitimacy on the research but do not get involved in the research process; second, responsive audience – where decisionmakers provide information, respond to researchers' queries and information needs, and are involved in most phases of the research process beyond conceptualization; and third integral partner – where there is a high degree of decisionmaker initiated contact and the decisionmaker is fully engaged as a significant partner, including in the conceptualization phase [2]. They also suggest four factors that influence the decisionmaker's role (stage of research process, time commitment required, alignment between decisionmaker expertise and research program, presence of an existing relationship). Their work provides a valuable starting point for considering how and when to best intertwine the research and decision-making processes.

Although interaction models of research utilization that bring researchers and decisionmakers together is one of the dominant models for promoting research utilization, there is little in the published literature about the conditions under which various interactive approaches are more or less successful. In addition, the literature is silent on the role of variation in the nature/attributes of research. For instance, does highly politicized or publicized research warrant different forms of interaction? Does descriptive research that provides evidence of problems require different or more interaction than intervention research that provides actionable evidence and solutions? Put differently, perhaps the nature of the research (*e.g*., certain attributes such as study politicization or stakeholder diversity) moderates the relationship between interaction and research utilization. In addition, the literature suggests that where there is decisionmaker involvement in research, decisionmakers should be included at all stages of the research process. However, early and sustained interaction can be onerous and costly, and it is not clear from the literature whether there may in fact be some cases where decisionmaker involvement is most appropriate only at the interpretation and dissemination stage (*e.g*., a retreat to older dissemination models). By looking at the impact and use of research findings by a heterogeneous group of decisionmakers for a specific, fairly publicized health services research initiative, our study provides some insights into these important areas.

When we judge the 'impact and use' of research findings in this paper, we do so in the context of work which outlines different kinds of research utilization. The utilization of research findings can be instrumental (a concrete application of research findings to make specific decisions or changes) and/or conceptual (*e.g*., change people's way of thinking) [9]. In addition, research can be utilized for more symbolic (*e.g*., political) purposes [10]. Indeed, the decision-making literature has documented the same uses for various kinds of information in the decision-making process [30]. Conceptual utilization is akin to the kind of "socialization" that Nonaka and Takeuchi [31] discuss as one way to convert or transfer tacit knowledge (knowledge that is difficult to formalize and communicate). In this process, socialization involves the exchange of tacit knowledge through joint, face-to-face activities "in order to produce some form of shared mental model... that can serve as a framework for moving forward" [5]. Landry *et al*. [8] suggest there is a need to look beyond narrow instrumental uses of knowledge, and Estabrooks [32] argues that the more indirect conceptual and symbolic uses for research are indeed an important and empirically demonstrated part of research utilization. Early work in this area suggests that symbolic use of research is far more common than instrumental use, which tends to be rare [33]. That research can be used in any or all of these three ways is consistent with the ideas that research and knowledge produce multiple effects rather than a single effect [11] and the utilization of research is therefore a process rather than a single event [29].

## Methods

In 2002, nearly $1 million was granted for the Canadian Adverse Events Study (CAES) [34]. The objective of CAES was to report on the incidence of adverse events in Canadian acute care hospitals based on a methodology used in similar studies in Australia [35] and elsewhere [36,37]. Although other international studies using the Australian methodology had fairly consistently demonstrated that roughly 10% of all hospital admissions resulted in an adverse event, approximately 50% of which were preventable, it was felt that Canadian data were needed to propel patient safety improvement activities in Canadian hospitals. In 2000, one of the agencies that funded CAES adopted knowledge translation as one of its priorities. Given the expected magnitude of the CAES findings, the agency undertook a knowledge translation effort of their own to try to generate a proactive response to CAES by a wide variety of national stakeholders. This effort involved holding two in-person forums on CAES while the study was being conducted and two web conferences, the first one four months prior to release of the study results, and the second one immediately prior to release. The agendas for the forums and web conferences focused on sharing results of various international studies CAES was trying to replicate, providing detailed methodology that would be used in CAES, holding break out group discussions about safety initiatives, and discussing media preparation for the CAES release. A diverse, national group of stakeholders was invited to attend the forums and web conferences including representatives from federal and provincial health ministries, professional organizations representing Canadian physicians and nurses, regulatory colleges governing various health professions, organizations servicing hospitals such as Canadian blood services, organizations representing health facilities and health executives, and safety-focused organizations such as the Institute for Safe Medication Practices Canada. To reiterate, the primary goal of the forums, was to stimulate proactive, instrumental use [10] of the CAES data as defined above.

We used a single case study approach, with embedded units, to assess the impact of this knowledge translation strategy. A case study design [38] was appropriate given our interest in learning not only about whether this knowledge translation strategy facilitated a response to CAES data, but also how it may have facilitated a response [39]. According to Yin, a case study "investigates a contemporary phenomenon within its real-life context, especially [suitable] when the boundaries between phenomenon and context are not clearly evident" [38]. In the present study, the case design permits the development of analytical insights where empirical results from the study can be used to support and inform theory–in this case related to models of effective decisionmaker involvement in research. Our approach is consistent with case study methods used in medicine and the social sciences [38,40].

The knowledge translation strategy–two forums and two webconferences–(hereafter referred to as "the forums") constitutes the case and data reported here are based on: semi-structured interviews conducted with forum participants, members of CAES research team, and organizers of the forums; observation of the forums and web conferences; and in-depth study of three of the stakeholder organizations embedded in the forum case. These three organizations, including a hospital, a representative organization for hospitals and health regions (with no official power), and an accreditation body, were chosen for their diversity with the idea that the forum process may impact different stakeholders in different ways.

### Data Collection

Semi-structured interviews were carried out with a random sample of forum stakeholders following the first forum held in June 2002, the second forum held in May 2003, and one month prior to release of CAES study results in May 2004. Following each forum, stakeholders who attended were grouped according to organization type (see Table 1) and one stakeholder from each category was randomly selected to complete an interview. Members of the CAES research team who attended the first forum were contacted for an interview following that forum, and all CAES researchers were contacted for an interview prior to the release of CAES. Interviews were also conducted with individuals in the two sponsor organizations that, together, funded CAES, planned, and hosted the forums. For the three organizations we studied in-depth, we visited the organization following each of the two forums, conducted two to three interviews with individuals involved in patient safety, and reviewed relevant documents outlining any patient safety initiatives.

**Table 1:** Forum Stakeholder Organization Categories

Organization Category	# represented at forum 1	# represented at forum 2	Examples
Professional	5	9	Canadian Medical Association
Service	6	4	Canadian Blood Services
Government	8	12	Saskatchewan Health, Health Canada
Organizational/Management Rep	5	10	Canadian Healthcare Association, Canadian College of Health Service Executives
Safety Focused	3	6	Institute for Safe Medication Practices, Canada
Representing other aspects of the health system	4	10	Consumers' Association of Canada
Other	5	11	Canadian Council for Health Services Accreditation
Total # of Organizations Represented	36	62	

Study interviews were conducted in person or over the telephone, depending on distance, by the principal investigator or the research assistant. In-person interviews lasted approximately 60 minutes and were recorded and transcribed. Telephone interviews lasted 30 to 45 minutes and relied on detailed field notes for analysis. Subjects were asked why they attended the forum, what they took away from the meeting, and whether they left feeling there was something more their organization could be doing in the area of patient safety. They were also asked to describe major patient safety initiatives in their organization and comment on the extent to which the forums may have played a role in their initiation. Ample opportunity for unstructured responses was provided.

### Analysis

All transcripts and field notes were coded using NVivo (2000). Coding was carried out with the original research questions in mind–the research questions guided a template analysis. Template analysis lies between content analysis where nodes are predetermined, and grounded theory where there are no predetermined nodes [41]. It is important to note that we were studying a researcher-decisionmaker interaction process that was unfolding as we were studying it–decisions about whether there would be in-person forums, web conferences, how many and when they would take place were made from one event to the next. Accordingly, the nature and timing of data collection, to some extent, had to emerge in response to forum activity. Following the two in-person forums we saw neither a high degree of explicit stakeholder response nor evidence of variation in responses by different stakeholders, including for the three organizations we were studying in-depth. Therefore, contrary to what we initially anticipated, a sub-analysis regarding whether the forums had a different impact on different kinds of stakeholders was not carried out.

## Results

In total, 33 interviews were conducted over the study period, 19 with stakeholders, 11 with researchers, and three with forum organizers. Of the stakeholders and researchers who were sampled systematically, 74% of the stakeholders randomly selected for an interview agreed to conduct an interview (nine of the ten stakeholders contacted following the first forum, and eight of 13 contacted following the second forum), and six of the eight researchers (75%) who attended the first forum agreed to be interviewed. Stakeholders who declined to participate in an interview tended to feel their organization was peripherally related to CAES, "since [organization name] is very much a peripheral stakeholder, I am sure that you would find the comments of other organizations to be far more relevant" (service organization stakeholder). Ten additional interviews were conducted using the more purposeful sampling approach described in the methods section: two additional stakeholders from the organizations we studied in a more in-depth fashion were interviewed prior to the release of CAES, five researchers who had some policy contact prior to release of CAES results were interviewed, and three of the individuals involved with designing and hosting the forums were interviewed. Together, the systematic and purposive sampling approaches yielded data from 33 interviews. The following three themes emerged from our analysis of these data. As noted, these themes were reflected across the diverse group of stakeholders that took part in the forum process. We have used quotes from study participants to help illustrate these themes [41].

### Forums promoted information sharing, discussion and networking, and consciousness-raising

The forums were perceived by respondents as being successful at informing stakeholders about CAES, as well as about other studies on the incidence of adverse events that have been conducted internationally. Our data also suggest that the forums contributed to broader learning about patient safety, "There was a real recognition that we are talking about something more than one clinician, there are bigger system issues that need to be looked at and how the management and the executives within those organizations can help, can find out specifically what those issues are and how they can start to move on them" (stakeholder, following the first forum). Some stakeholders were critical of the forum process for being too researcher driven with little room for input (methodological and otherwise) from stakeholders, "there was a kind of command and control feel to it and what we needed was more of a participative, action approach going forward". Others identified a more bidirectional nature of the forum exchange, "the forums provided an opportunity for researchers to see and hear contextual issues in the environment as they are framing up their research"..

The forums provided opportunities for stakeholders to network with one another and find out what others were doing in the area of patient safety–people seemed to find value in knowing what others were doing and used that information as a gauge for where their organization should be. The forums also stimulated discussion that lasted beyond the forums. One stakeholder noted that, "in our organization we have now tried to discuss the key things that we must address when the study data are released." One researcher stated that, "since the forums, I have had contact with a number of people in several provinces... and mostly, the contact is around questions about the nature of the study and when it is going to appear. Sometimes specific questions about the kinds of information that will be reported arose."

The forums clearly helped to bring CAES (and patient safety more generally) into stakeholder consciousness, and onto "people's mental agenda if not on their organization's agenda" (researcher, following the first forum). Data suggest the forums went even further than consciousness-raising and helped to create some sense of urgency for action in the area of patient safety, "The aim [of the forum] was to say to those of us involved in patient safety to beware and prepared so that we won't be shocked when the data come out... to sensitize us and get some preparation." Another stakeholder stated that, "I think the importance of this forum is that it was really based on introducing the Baker-Norton study [CAES] and the fact that it's a reality, it's going happen, we are going to have data and, for us, I guess, the urgency of dealing with our own responses and potential activities became more of a reality" (stakeholder, following the first forum).

### Forums promoted less instrumental research utilization

In addition to the informational, consciousness-raising impact of the CAES forums described above, the architects of the CAES forums were interested in promoting a somewhat more proactive, instrumental use of CAES research. Following the first forum, one of CAES/forum sponsors suggested that, "At future meetings we could get the stakeholders to give a synopsis re strategies their organizations are taking around adverse events with the aim of making it their agenda where we become players in a forum of affected people." However, as one stakeholder stated following the second forum, "The original intent [of the forum] was to help prepare stakeholders for a coordinated response... I'm not sure we ever got there. I think that may have been a lot to expect... we never even got to any coordinated communication strategy." Interviewees were asked about new and ongoing patient safety initiatives and the extent to which these initiatives were driven by the forums. Consistent with the data already described, responses suggest the forum approach was useful for getting CAES on the agenda of a wide range of Canadian stakeholders, and perhaps facilitating other safety initiatives rather than creating them, "I think that the forums have been sort of a starting point, there have been other initiatives and other concerns that we have been able to put forward and go maybe faster with because of this. Would we be at the same point today? Probably not. Would we be going down that route? Probably. So it's been a valid contributor to the speed at which we have taken things" (stakeholder, following the second forum).

### Meeting the needs of decisionmakers was challenging

One of the most prominent themes, identified by stakeholders and researchers following the first forum, was that the objectives of the forum were not well understood. As one stakeholder stated, "I don't think the objectives were clear. We all went with mixed messages. There has been so much discussion of patient safety that people probably went expecting different things... mid-morning I pulled out my invitation and had to read it again." This theme is important as we try to understand what decisionmakers want or, perhaps more pointedly, feel they need from this type of interaction. The perceived lack of clarity of the forum objectives may reflect the challenges associated with convening an exchange forum where there are several different types of stakeholders, not to mention researchers and sponsors, each with their own priorities. In the face of unclear objectives, our interviews revealed that the methodologists came to the forum looking for input into the study methodology while stakeholders representing health service executives and health facilities came hoping to obtain direction and tools for addressing patient safety problems.

The second forum was more clearly aimed at showcasing the patient safety initiatives and challenges of visible stakeholders like the Canadian healthcare accreditation body, Canadian Medical and Nursing Associations, and other key stakeholders, as well as helping organizations develop a media response to the CAES results. The media session included a presentation featuring one organization's media experience with a critical health related issue, and was followed by breakout groups designed to identify target audiences for a communication plan, outline key messages, etc. Our interview data, along with data from a small number of completed forum evaluations and our observations of the second forum suggest that people did not feel able to take away tools or strategies for addressing adverse events, and the media preparedness component of the second forum was not widely seen as valuable. Consistent with the literature [1,16], nearly all stakeholders expressed an interest in receiving interim study results before the final release, even though it was made clear that would not be possible given prior agreements between the CAES researchers, forum funders, and the Canadian Medical Journal (CMAJ) that had agreed to fast track peer review and publication of the study results.

Our data indicate that stakeholders want "just-in-time" data and operate on different timelines than the researchers or the forums [42]. The bulk of the exchange activity took place during the first two forums, held two years and one year prior to the release of the CAES results. The two web conferences, which lasted approximately an hour and provided only brief study updates, were held four months and one month prior to release. Stakeholders were most interested in release of the results and felt that there was too much time between the forums and release. Following the first forum, one stakeholder stated, "We're not ready yet to hear what you want to tell us in terms of the next step, we've got a million other issues that are before us, this [CAES] will become an issue when we're much closer to it hitting the media... until then our members aren't ready to hear it and we have other priorities." Finally, stakeholders were also clear in their interest for more follow-up and direction at the time of data release. Similarly, members of the research team felt this would be beneficial, "If there was another forum, it should be about implications or meaning of the results. What does it mean? What should we do? Implications around actions, maybe propose/present what other jurisdictions (UK, Australia, etc) have done with results and how they have improved patient safety based on the results of this study. It would be helpful for managers to know what the data mean and what to do with it."

Despite this seemingly high degree of unmet need, stakeholders reported that they would continue to attend or take part in additional forums or web conferences related to CAES. Indeed, the number of stakeholders showing continued interest in CAES and the forum process rose throughout the forum initiative, with 36 organizations represented at the first forum, 62 at the second forum and an even higher number on the two subsequent web conferences.

## Discussion

The knowledge translation forums and web conferences we studied were perceived to be successful at providing information about CAES and focusing stakeholders' attention on patient safety and rates of adverse events in Canadian hospitals. The forums also seemed to stimulate discussion and awareness, and help prepare a key group of national stakeholders to, in some way, receive what were certain to be highly sensitive study results. This was accomplished in a positive light by highlighting that system changes would be the likely target for change in response to the study. Finally, the forums created some sense of urgency around addressing patient safety issues. The forums were seen as less successful at helping stakeholders prepare for media queries emerging from CAES, stimulating any kind of unified or proactive response to the results, or aiding stakeholders with study interpretation or next steps that could be used to try to reduce rates of adverse events and improve patient safety. These findings were consistent for a variety of stakeholders working in different health related and policy settings. Broadly, our results are consistent with the ideas that research and knowledge produce multiple effects rather than a single effect [11].

If we return to the three ways in which research can be 'used', where instrumental use involves concrete application of research findings to make specific decisions or changes, conceptual use has to do with changing people's way of thinking) [9], and symbolic use reflects political uses of research findings [10], our data suggest that the forums had more of a 'conceptual' and 'symbolic' impact than the 'instrumental' impact that was the primary aim of the forums. Judged against literature that suggests it is important to move beyond looking at narrow, instrumental uses of research [8], and literature arguing that conceptual and symbolic uses for research are indeed an important and empirically demonstrated aspect of research utilization [32], the forum approach we studied seemed to have a positive impact. Similarly, if research is seen not as a product for problem-solving, but "a process of argument or debate to create concern and set the agenda" [43], our results suggest that despite their shortcomings the forums may have successfully helped to shape decisionmaker values and stance toward safety and adverse events, thereby allowing decisionmakers to see these potentially threatening study data as constructive, rather than destructive. These effects are important because for research to have an impact it must be consistent with decisionmakers' values [17,43]. That said, from the perspective of improving quality and safety, there remains a clear need to move further and faster with substantive changes and improvements in this critical area of improving patient safety.

Consistent with the literature on interaction approaches to research and knowledge utilization, the more social, face-to-face, interactive aspects of the forums had a positive impact, albeit predominantly conceptual and symbolic, on stakeholders in terms of propelling them in the direction of patient safety. Also consistent with the literature, our data suggest that the impact of the forums might have been strengthened (and perhaps more instrumental), had the exchange process been extended beyond release of the study results in a way that would have brought stakeholders and researchers together to interpret the results and discuss next steps for addressing patient safety problems. Indeed, as noted in our review of the literature, this joint interpretation aspect is seen as a critical component of the interaction approach to research utilization [4,5,15]. Future endeavors to bring researchers and decisionmakers together should ensure that interactions include interpretation of the study results, particularly for studies where the results may be more tacit [44], and direction for action may not be immediately clear or obvious from the study findings.

### Implications for interaction in knowledge translation

Our results contribute to the knowledge translation literature by suggesting some conditions under which various interactive approaches to research utilization may be more or less successful. For instance, our findings are not consistent with literature stating that early and prolonged contact between researchers and decisionmakers is critical for successful research utilization [15,18,19,29]. Instead, our case analysis encourages us to consider whether, for studies with multiple stakeholders, fixed methods (*e.g*., replication studies) or highly public or politicized findings, interaction approaches that focus around the latter part of the study period and release of the study results may be more appropriate and effective. Each of these research attributes (multiple stakeholders, fixed methods, high public interest) on its own, or together, may require more targeted interactions that do not necessarily span the length of the research process–multiple stakeholders may have competing or inconsistent priorities that may complicate an already challenging exchange process; fixed questions and methods characteristic of replication studies leave no room for decisionmaker input into early aspects of research; and highly publicized studies are likely to attract interest regardless of whether there was prior involvement in the process.

This case study raises several questions and, in doing so, points out the need for research that further addresses questions of: when and under what conditions decisionmaker involvement in research is warranted; and, where it is warranted, under what conditions is involvement most appropriate for various stages of the research process. As Ross *et al*. note, there is a need "to be strategic about involving decisionmakers in the research process... a one-size-fits-all approach holds little chance of success... picking and choosing whether and how to involve decisionmakers", among other things, is a critical step in the process [2]. The differing timeframes within which researchers and decisionmakers operate that came through in our data, and the fact that many research studies run longer than an organization's strategic plan and the average decisionmakers' tenure, further underscore the need to be efficient and strategic when it comes to planning researcher-decisionmaker interactions.

The current case study also leads us to question whether and/or when there has to be pre-existing concordance in researcher and decisionmaker interests that is often cited in the literature [16]. Indeed, it is possible that with highly public studies, the nature of the study itself, together with a broad based forum process (strategically designed to address concerns, allay fears, and promote a productive response), may actually bring about alignment of researcher – decisionmaker interests. In the case of CAES, by researching a highly politicized topic such as adverse event rates in Canadian hospitals, the researchers seemed to help set a common agenda and focus on this area. A year after the release of the CAES results, a Canadian campaign to intervene to reduce adverse events was undertaken by some of the CAES investigators. Decisionmakers in hospitals and health regions from across Canada have signed up for the campaign *en masse*, committing substantial organizational resources to improving patient safety in certain areas [45]. Of course it is also possible that, for the case we studied, this alignment of researcher – decisionmaker interests around patient safety was facilitated by other high-profile patient safety reports, starting with the Institute of Medicine reports, To Err is Human [46] and Crossing the Quality Chasm [47] in addition to CAES.

Finally, we question whether interaction can successfully align unaligned interests, or resolve value conflicts between decisionmakers and researchers, as the interaction model implies. These kinds of barriers to research utilization are not only pervasive but often insurmountable, even when there has been prolonged interaction [22]. Decisions concerning the value of interaction need to take account of the nature, goals, and potential of the interaction, if interaction is going to be useful in complex research and practice contexts.

So, in addition to the three models of decisionmaker involvement (formal support, responsive audience, and integral partner) articulated by Ross *et al*. [2] and noted in the literature section, the current case study suggests a fourth model of decisionmaker involvement for consideration and future research called "intermittent partner" (particularly for preparation for use of results). In terms of the four factors that Ross *et al*. [2] suggest influence the decisionmaker's role (stage of research process, time commitment required, alignment between decisionmaker expertise/research program, presence of an existing relationship), we propose further exploration of a fifth factor that might influence the decisionmaker's role that has to do with the nature or attributes of the research topic and findings. Future research could test whether the four attributes illuminated by the current case study – stakeholder diversity, addressability/actionability of results, finality of study design and methodology, and politicization of result – or other attributes, can be used to specify some of the conditions under which different approaches to interaction in knowledge translation are likely to facilitate research utilization (instrumental, conceptual and/or symbolic). For instance, stakeholder diversity may be an important moderator variable such that interaction processes may explain a significant amount of variation in instrumental research utilization under conditions of low stakeholder diversity, but not under conditions of high stakeholder diversity (perhaps because a homogeneous stakeholder group permits greater focus on a small number of highly relevant issues germane to the study and to implementation of change by that group). Another example may be that studies with politicized results moderate the relationship between sustained interaction and conceptual research utilization where, under conditions of high politicization, this relationship is more significant. Additional research on these and other questions, that uses different research designs would contribute to greater understanding of the relationship between interaction processes and the outcome of research utilization, including potential moderator variables.

### Broader implications for knowledge translation research

Even studies with discrete outcomes, such as a systematic review of the effectiveness of a specific treatment in a certain clinical situation, face considerable challenges when it comes to successful knowledge transfer and uptake [48,49]. Our case study examined a knowledge translation effort for research with broad implications, multiple target audiences, and potentially inflammatory results that underlined the need for change, but offered no clear direction for achieving it. Our analysis suggests that the response to this startling data (by extrapolation the CAES data showed that somewhere between 9,000 and 24,000 deaths from adverse events could have been prevented in Canadian hospitals in the year 2000 [34]) was at most symbolic and conceptual.

It is clear that in the face of evidence, pervasive barriers to change exist in the contexts of clinical, policy, and organizational decision-making. The barriers are often different in each of these arenas, but include traditions of autonomy, politics and values, competing priorities, perverse financial incentives, and lack of knowledge of what and how to change, respectively. This situation further underscores the need for additional research in the area of research utilization. A theory of research utilization must continue to look beyond interaction approaches and also consider models of change. For instance, the organizational literature suggests that the kind of incremental change that is inherent in current interaction models, by and large, fails to lead to any meaningful organizational reorientation because incremental change is not forceful enough to overcome cognitive and motivational inertia [50,51] or the stabilizing effects exerted by scientific communities. Incorporating more radical models of change [52] from the organization arena into a theory of research utilization may bode well for expediting scholarly work in this important area.

Work on research utilization in healthcare might also do well to draw on work in the organizational literature on learning capacity. This work focuses on the user context and the ability to recognize the value of new knowledge and apply it in the organization [53]. Though a focus on learning capacity may seem reminiscent of the older, now abandoned research pull models and certain elements of the dissemination model [14], it may be fruitful to revisit some of the promising levers for change that may exist in both the researcher and user contexts, in addition to further examining the sphere of researcher-decisionmaker interaction and research utilization more broadly. It may also be instructive to revisit the work of Argyris and colleagues [54] concerning action science and Torbert's more recent work on action inquiry [55]. This work links learning capacity to a well-established participatory action theory and might strengthen current interaction models in research utilization.

Finally, it is important to address not just the inherent plausibility and demonstrated validity of research utilization theory, but the circumstances in which it may or may not be applicable. There are important counter-examples to the voluntary and incremental theory of change in health care, *e.g*., the transformations of the National Health Service in the UK and the Veterans Affairs health system in the US, and changes in use of hormone replacement therapy in menopausal women. The latter was largely research-driven but did not involve early-stage or sustained interaction among researchers and decisionmakers; in fact the decisionmakers were as much the public as their physicians. In the former two cases, the change was policy-driven and mandated, and accompanied by structures and lines of authority that greatly accelerated the pace of reform. A full account of how major and rapid change occurs would both refine the theory of research utilization and an understanding of where it is central or tangential to improvement, and where and when other options present better alternatives.

The strengths and limitations of this study are, to some degree, tied up with the strengths and weaknesses of the knowledge translation effort we studied. For instance, one strength of the forum approach is that it targeted stakeholders at several levels of the health care system, and in doing so, recognized the importance of the organizational environment, social, regulatory, and other contexts in the research translation process [56-58]. In parallel, one strength of our study is that we were able to look at the impact of a knowledge translation effort that took context into account. A limitation of the approach we studied lies in its failure to sustain interaction at what decisionmakers saw as the most critical juncture of the study–the release and interpretation of the results and discussion of next steps. Correspondingly, we were unable to look at the impact forum interactions might have had, had they been carried out in these important latter stages of CAES. An additional limitation of this study has to do with our inability to more fully examine the different perceptions and expectations of stakeholders versus researchers, including the extent to which these differing perspectives may be important determinants of successful interaction.

## Conclusion

Current interaction theory proposes early and sustained interaction between researchers and decisionmakers as mechanisms for improving research utilization. The current study adds to this literature by raising questions about whether there are some conditions under which full scale interaction and decisionmaker involvement in research will be more or less effective. Future research could test whether four attributes illuminated by the current case study (stakeholder diversity, addressability/actionability of results, finality of study design and methodology, and degree of politicization of results) can be used to specify some of the conditions under which different approaches to interaction in knowledge translation are likely to facilitate research utilization. This study examined a knowledge translation effort in its natural context. We were fortunate to be able to focus on the interaction component of research utilization, a component which we suggest requires further exploration. We were able to examine a knowledge translation effort that required complexity in order to manage the fact that CAES would be highly publicized, and the results would warrant change at so many levels. These complexities help us to suggest certain conditions under which more targeted researcher-decisionmaker interactions may be most effective. These complexities also enable us to see the value in extending our search so we can specify a broader theory of research utilization that accounts for the slow pace of change in this area.

## Competing interests

The author(s) declare that they have no competing interests.

## Authors' contributions

LG lead the design and implementation of the study, engaged in data collection, analysis and drafting of the manuscript. SL and AC contributed to study design, data interpretation and helped to draft the manuscript (SL and AC were LGs postdoctoral supervisors when this study was conducted). LZ contributed to data collection and analysis. All authors read and approved the final manuscript.
